# Mycobacterium tuberculosis Lineages Associated with Mutations and Drug Resistance in Isolates from India

**DOI:** 10.1128/spectrum.01594-21

**Published:** 2022-04-20

**Authors:** Siva Kumar Shanmugam, Narender Kumar, Tamilzhalagan Sembulingam, Suresh Babu Ramalingam, Ashok Selvaraj, Udhayakumar Rajendhiran, Sudha Solaiyappan, Srikanth P. Tripathy, Mohan Natrajan, Padmapriyadarsini Chandrasekaran, Soumya Swaminathan, Julian Parkhill, Sharon J. Peacock, Uma Devi K. Ranganathan

**Affiliations:** a Indian Council of Medical Research (ICMR)-National Institute for Research in Tuberculosis, Chennai, India; b Department of Medicine, University of Cambridge, Addenbrooke’s Hospital, Cambridge, United Kingdom; c World Health Organization, Geneva, Switzerland; d Department of Veterinary Medicine, University of Cambridge, Cambridge, United Kingdom; University of Manitoba

**Keywords:** drug resistance, *Mycobacterium tuberculosis*, lineage, whole-genome sequencing

## Abstract

Current knowledge on resistance-conferring determinants in Mycobacterium tuberculosis is biased toward globally dominant lineages 2 and 4. In contrast, lineages 1 and 3 are predominant in India. In this study, we performed whole-genome sequencing of 498 MDR M. tuberculosis isolates from India to determine the prevalence of drug resistance mutations and to understand the genomic diversity. A retrospective collection of 498 M. tuberculosis isolates submitted to the National Institute for Research in Tuberculosis for phenotypic susceptibility testing between 2014 to 2016 were sequenced. Genotypic resistance prediction was performed using known resistance-conferring determinants. Genotypic and phenotypic results for 12 antituberculosis drugs were compared, and sequence data were explored to characterize lineages and their association with drug resistance. Four lineages were identified although lineage 1 predominated (43%). The sensitivity of prediction for isoniazid and rifampicin was 92% and 98%, respectively. We observed lineage-specific variations in the proportion of isolates with resistance-conferring mutations, with drug resistance more common in lineages 2 and 3. Disputed mutations (codons 430, 435, 445, and 452) in the *rpoB* gene were more common in isolates other than lineage 2. Phylogenetic analysis and pairwise SNP difference revealed high genetic relatedness of lineage 2 isolates. WGS based resistance prediction has huge potential, but knowledge of regional and national diversity is essential to achieve high accuracy for resistance prediction.

**IMPORTANCE** Current knowledge on resistance-conferring determinants in Mycobacterium tuberculosis is biased toward globally dominant lineages 2 and 4. In contrast, lineages 1 and 3 are predominant in India. We performed whole-genome sequencing of 498 MDR M. tuberculosis isolates from India to determine the prevalence of drug resistance mutations and to understand genomic diversity. Four lineages were identified although lineage 1 predominated (43%). The sensitivity of prediction for isoniazid and rifampicin was 92% and 98%, respectively. We observed lineage-specific variations in the proportion of isolates with resistance-conferring mutations, with drug resistance more common in lineages 2 and 3. Disputed mutations (codons 430, 435, 445, and 452) in the rpoB gene were more common in isolates other than lineage 2. Phylogenetic analysis and pairwise SNP difference revealed high genetic relatedness of lineage 2 isolates. WGS based resistance prediction has huge potential, but knowledge of regional and national diversity is essential to achieve high accuracy for resistance prediction.

## INTRODUCTION

According to the Global Tuberculosis (TB) Report 2020, an estimated 10 million people were diagnosed with TB in 2019. In the same year, close to half a million people developed rifampicin-resistant TB (RR-TB), of which 78% had multidrug-resistant TB (MDR-TB) (1). An important contributor to morbidity and mortality from TB is resistance to first or second-line anti-TB drugs (1).

Despite global efforts toward tuberculosis management and control, just one-third of patients who developed MDR- or RR-TB received treatment in 2018 (1). In India where the burden of MDR/RR-TB is the highest in the world, 79.8% of patients with MDR/RR-TB were started on treatment (2). Among patients on MDR-TB treatment in India, only 39.5% were successfully treated (2). Action to address this includes the use of rapid molecular methods for drug susceptibility testing as opposed to time-intensive culture-based phenotypic testing to determine optimized treatment regimens.

There has been major progress over the last few years with the scale-up of WHO-approved rapid molecular diagnostic technologies, such as the Xpert MTB/RIF assay and line probe assay (LPA) (3). The Xpert MTB/RIF is a rapid molecular assay that can be used close to the point of care by operators with minimal technical expertise, enabling diagnosis of TB and simultaneous assessment of rifampicin resistance to be completed within 2 h (3). Line probe assays (LPAs) are rapid molecular diagnostics that can detect M. tuberculosis and drug resistance. Although LPAs are more technically complex (designed for reference or regional laboratory settings) and take longer to perform than the Xpert MTB/RIF assay (Cepheid, Sunnyvale, CA, USA), they detect isoniazid (INH) resistance in addition to rifampicin (RIF) resistance. LPAs detect RIF and INH resistance by identifying mutations in the *rpoB*, *katG*, and *inhA* genes (4). Despite being rapid, several studies have shown variable accuracy of currently available molecular methods in detecting resistance to anti-TB drugs, particularly for second-line anti-TB drugs (5). This is mainly due to gaps in our understanding of phenotypic resistance and its causative genetic determinants.

Supplementary to this is to understand the predominant TB lineages and their associations with drug resistance. Different genetic lineages have been associated with variation in MICs of antituberculosis treatment (ATT) drugs, acquisition of mutation profiles, and fitness cost of resistance-conferring mutations (6). Most of our current understanding of the genetic determinants of drug resistance comes from studies of the globally dominant lineages 2 and 4 (7). However, M. tuberculosis lineages prevalent in India are markedly different from the rest of the world, with a predominance of lineage 1 in the south and lineage 3 in central and northern regions (7, 8). Currently, there are a limited number of studies on the whole-genome sequencing (WGS) of M. tuberculosis isolates and the genetic determinants of drug resistance from India (9–11). In our previous report from Tiruvallur, India (12) lineages 2 and 3 had a strong association with drug resistance. Furthermore, lineage association, frequency, and type of gene mutation vary between different geographical regions in India and other parts of the world (13).

In this study, we performed WGS on 498 MDR M. tuberculosis isolates from Southern India. We used known genetic determinants to predict drug resistance and compared this with phenotypic test results for 12 antituberculosis drugs. We determined the prevalence of drug resistance mutations among different lineages. A phylogenetic analysis combined with whole-genome pairwise SNP difference was used to determine the genetic diversity among the isolates belonging to different lineages.

## RESULTS

### Bacterial collection.

**FIG 1:**
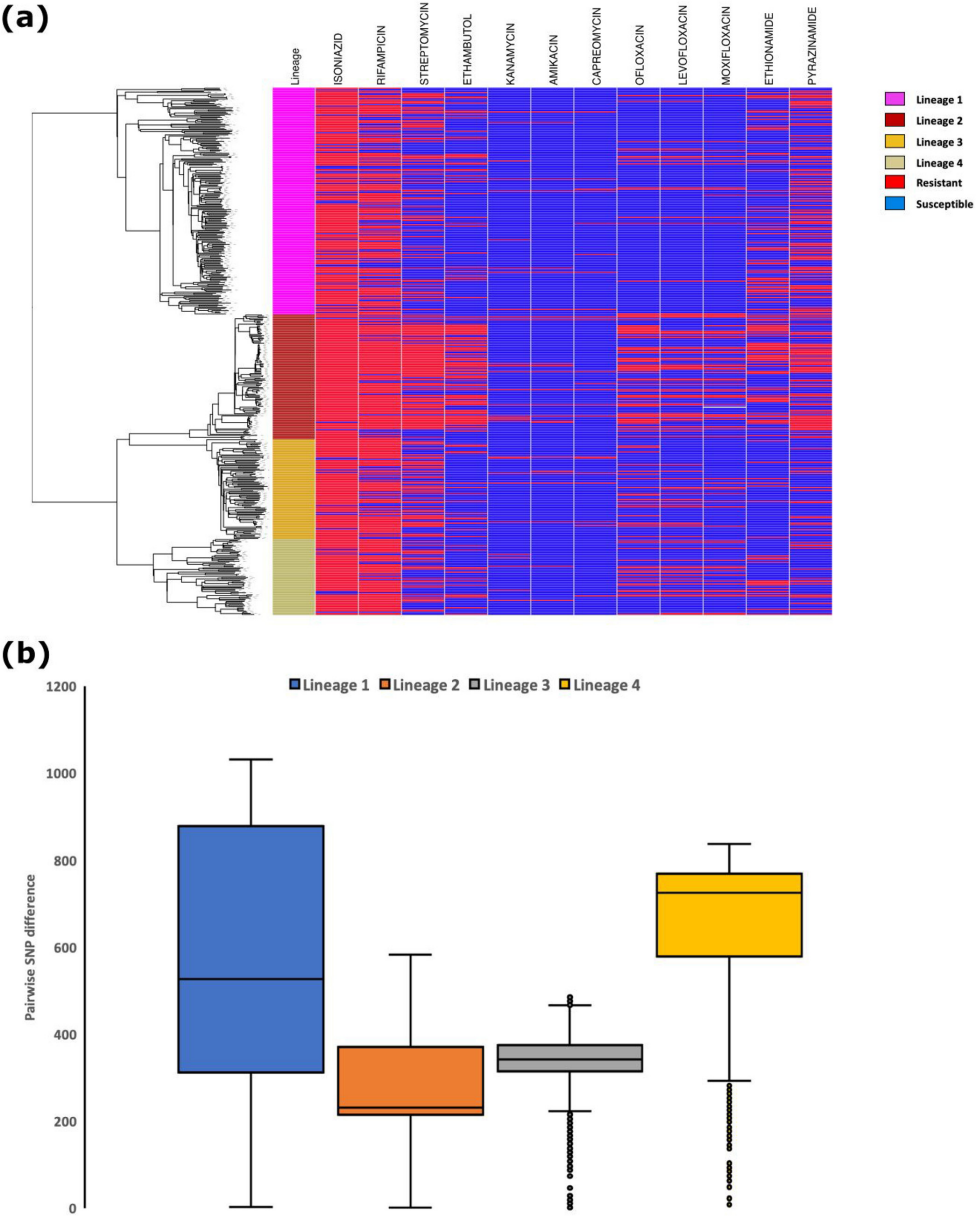
(a) Phylogenetic tree of 486 isolates and phenotypic drug susceptibility results. (b) Pairwise SNP differences for the four lineages.

Phenotypic drug susceptibility results were available for 495 of the 498 sequenced isolates, of which one isolate was identified as Mycobacterium avium by genome analysis and was excluded from further analysis. The remaining isolates (*n* = 494, Table S1) were from suspected MDR-TB patients, and the majority (87%, 429/494) proved to be MDR (including rifampicin-resistant isolates) of which 19 were identified as previously extensively drug-resistant tuberculosis (pre-XDR) (3.8%, 19/429) (Fig. 1A). The collection was genetically diverse and belonged to lineage 1 (43%, 211/494), lineage 2 (23%, 116/494), lineage 3 (19%, 93/494) and lineage 4 (14%, 71/494). In addition, there were 3 cases (0.6%) of mixed lineages with lineage 2 being common in all cases: lineage (1 + 2), lineage (2 + 3), and lineage (2 + 4). Drug resistance was more common among isolates in lineage 3 and 2, with 87% (81/93) and 85% (99/116) being either rifampicin resistance or MDR, respectively (Fig. 1). Similarly, the proportion of isolates that were pre-XDR was higher among lineage 2 (24%, 28/116) and lineage 3 (21%, 20/93). Of the 19 (3.8%) isolates that were classified as pre-XDR-TB, 8, 6, 4, and 1 were lineage 2, lineage 1, lineage 4, and lineage 3, respectively.

### First-line drugs.

For isoniazid, there were 406 of 445 phenotypically resistant isolates (91%) had at least one known resistance-conferring variant in *katG*, *inhA,* or *fabG1*. The predominant mutation was p.S315X in *katG*, (328/406, 81%). The second most common mutation n.C-15T in *fabG1* was detected in 68/406 (17%) isolates, of which just under half (*n* = 31) also had the p.S315T mutation. Of the 5 isolates that were phenotypically susceptible despite having one of the predominant mutations, 3 had p.S315X and the other two had either n.C-15T in *fabG1* or an indel in *katG*. These isolates likely represent errors in phenotyping.

For rifampicin, 367 of 375 phenotypically resistant isolates (98%) had at least one resistance-conferring mutation in *rpoB*. The predominant mutation was p.S450X (247/367 [67%] isolates).

**FIG 2:**
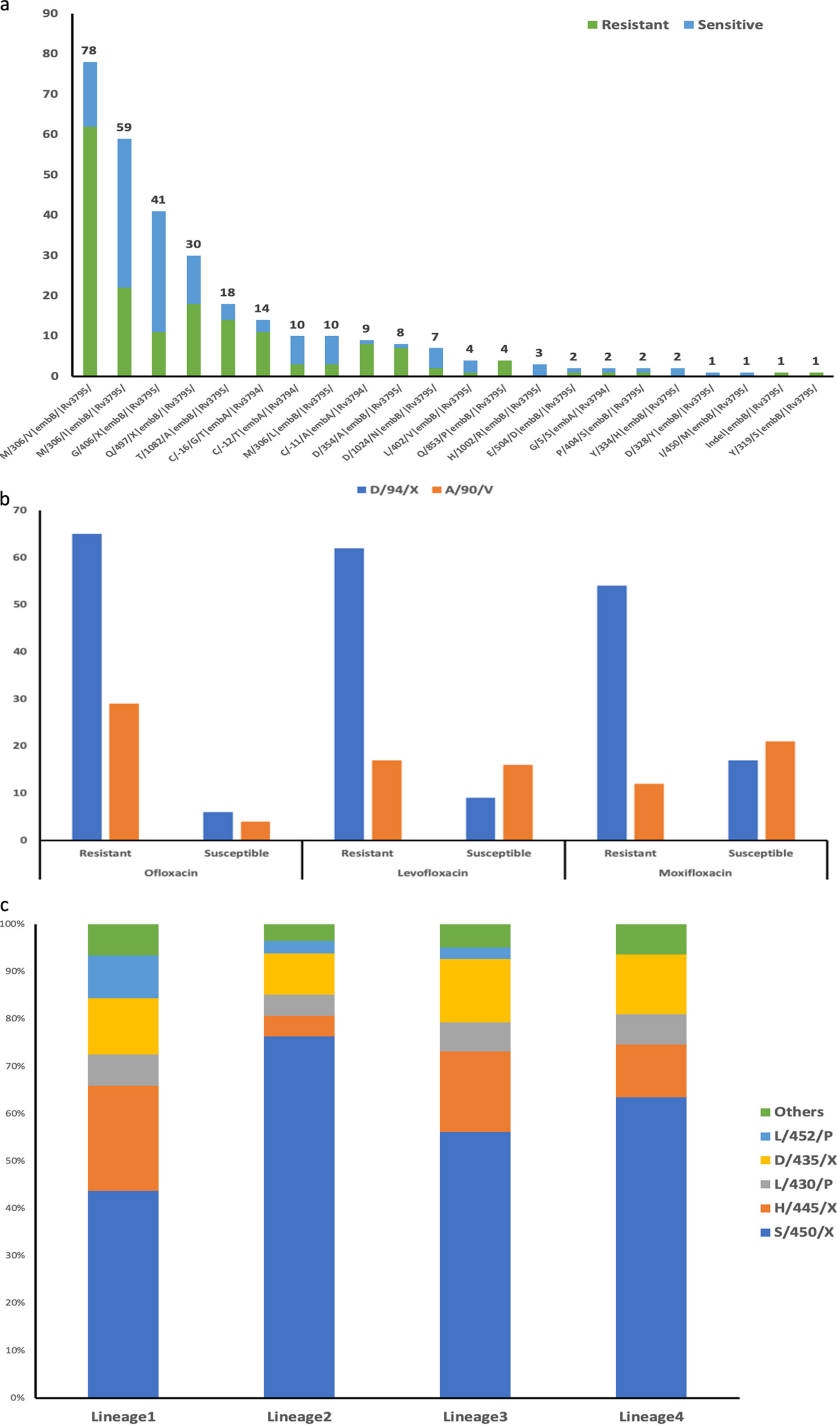
Genetic determinants of resistance. (a) Genetic determinants in isolates that were phenotypically resistant and susceptible to ethambutol. (b) Frequency of p.D94X and p.A90V mutations for isolates that were phenotypically resistant and susceptible to three fluoroquinolone drugs. (c) Distribution of genetic determinants of resistance to rifampicin in the four lineages.

For ethambutol, 126 of 132 phenotypically resistant isolates (95%) had at least one resistance-conferring mutation in *embA* or *embB*, the most frequent being p.M306X (87/126 [69%] isolates). Mutations in *embB* and *embA* have been strongly associated with increases in MICs over a wide range (14). However, 35% (126) of phenotypically susceptible isolates (362) had a resistance mutation. Together, there were 22 different mutations identified among 62% (307/494) isolates irrespective of their resistance phenotype. The proportion of isolates that were phenotypically resistant or susceptible varied across these mutations as shown in Fig. 2a.

For pyrazinamide, 119 of 181 phenotypically resistant isolates (66%) had one or more mutations identified in *pncA*. A total of 69 different mutations were identified, with no mutational hot spots for resistance-causing mutations, which is consistent with previous studies (15, 16).

### Second-line drugs.

For streptomycin, 148 of 226 phenotypically resistant isolates had at least one resistance-conferring mutation in the *rrs* or *rpsL*. The most frequent mutation p.K43R in *rpsL* (99/148 67%) was followed by p.K88R (14/148 9.4%) in *rpsL* and n.C517T (14/148 9.4%) in *rrs* genes. For aminoglycosides, 42 isolates were phenotypically resistant to at least one of the three aminoglycosides tested (amikacin [AMIK]-32, capreomycin [CPR]-24, and kanamycin [KAN]-26), of which 25 had at least one resistance-conferring mutation identified (AMIK-15, CPR-16, and KAN-19). The most frequent mutation was n.A1401G in *rrs*, identified in 14 (93%, AMIK), 11 (46%, CPR), and 13 (68%, KAN) among phenotypically resistant isolates. Small indels in *tlyA* were also identified in 3 (12%) of the phenotypically CPR-resistant isolates.

For fluoroquinolones, 116 isolates were phenotypically resistant to one of the three fluoroquinolones tested (levofloxacin [LEV]-76, moxifloxacin [MOX]-89, and ofloxacin [OFX]-111), of which 104 had at least one resistance-conferring mutation identified (LEV-83, MOX-76, and OFX-102). The most common mutations across the three drugs were p.A90V and p.D94X in *gyrA*. These mutations were also identified in phenotypically susceptible isolates, as shown in Fig. 2b. The mutation p.A90V was comparatively less frequent in OFX susceptible isolates compared to LEV and MOX.

For ethionamide, 70 of the 124 phenotypically resistant isolates had one or more resistance-conferring mutations in *inhA*, *ethA*, or *fabG1*. The most common mutation was n.C-15T in *fabG1* 53/70 isolates, (75%). There were 17 (53%) isolates in which n.C-15T mutation was identified but the phenotype was susceptible. This indicated the difficulty in performing the phenotypic testing for ethionamide as reported previously (17).

### Phenotype-genotype comparison.

Phenotypic drug susceptibility based on genotypic predictions for all 494 isolates were compared with phenotypic drug susceptibility testing results for 12 antituberculosis drugs (Table 1). Concordance for each anti-TB drug ranged from 81.5 to 96.8% for first-line and second-line drugs, except for ethambutol which had a concordance of 72%. Sensitivity for first-line antituberculosis drugs ranged from 65.8% (pyrazinamide [PZA]) to 97.9% (rifampicin [RMP]) and specificity ranged from 47.9% (RMP) to 93.5% (PZA). Sensitivity for second-line drugs ranged from 56.4% (ethionamide [ETH]) to 93.4% (MOX) and specificity ranged from 88.3% (MOX) to 99.6% (AMIK). Sensitivity for resistance prediction for fluoroquinolones (FLQ) ranged between 92 and 93% (92% for ofloxacin [OFX], 93% for LEV, and 93% for MOX). Details of the major genetic mutations detected for the 12 drugs are listed in Table 2.

**TABLE 1:** Summary of phenotypic susceptibility tests and genotypic predictions for 494 isolates

Category/drug	True positive^*a*^	True negative^*b*^	False positive^*c*^	False negative^*d*^	Sensitivity (%)	Specificity (%)
First-line						
Isoniazid	409	44	5	36	91.9	89.8
Rifampicin	367	57	62	8	97.9	47.9
Ethambutol	126	236	126	6	95.5	65.2
Pyrazinamide	119	292	21	62	65.8	93.3
Second-line						
Streptomycin	148	258	10	78	65.5	96.3
Kanamycin	19	453	9	13	59.4	98.1
Amikacin	15	466	2	11	57.7	99.6
Capreomycin	16	465	5	8	66.7	98.9
Ofloxacin	102	365	18	9	91.9	95.3
Levofloxacin	83	368	37	6	93.3	90.9
Moxifloxacin	71	369	49	5	93.4	88.3
Ethionamide	70	338	32	54	56.5	91.4

a True positive, phenotypically resistant, and contained known resistance-conferring mutations.

b True negative, phenotypically susceptible, and no known resistance-conferring mutations.

c False positive, phenotypically susceptible but known resistance-conferring mutations.

d False negative, phenotypically resistant but no known resistance-conferring mutations.

**TABLE 2:** Predominant genetic mutations (present in at least 5 isolates) identified in 494 isolates

First-line drugs			Second-line drugs		
Drug	Gene	Mutations^*a*^	Drug	Gene	Mutations^*a*^
Isoniazid	*katG*	p.S315X, Indels	Aminoglycosides	*rrs*	n.A1401G, Indels
	*fabG*1-promoter	n.C-15T, n.T-8C	Ethionamide	*fabG*1- promoter	n.C-15T, n.T-8C
	*inhA*	p.S94A		*inhA*	p.S94A, p.I21T
Rifampicin	*rpoB*	p.S450X, p.L430P, p.H445X, p.D435X, p.Q432L, Indels		*ethA*	Indels
Ethambutol	*embB*	p.M306X, p.Q497R, p.T1082A, p.G406X, p.D354A, p.D1024N	Fluoroquinolones	*gyrA*	p.A90V, p.D94X,
	*embA*	n.C-12T, n.C- 16×	Streptomycin	*rpsL*	p.K43R, p.K88R
Pyrazinamide	*pncA*	Indels, p.G132A, p.L27P, p.I5S, p.T76P		*rrs*	n.A1401G, n.C517T, n.A514C

a Amino acid changes are represented with the prefix “p” and nucleotide changes with the prefix “n”. The letter “X” indicates codons/nucleotide positions where more than one change was observed. “Indels” refer to small insertions and deletions observed in the genes.

### Lineage-specific observations.

We observed lineage-specific variations in the proportion of isolates with resistance-conferring mutations. For rifampicin, the p.S450X mutation was highest for lineage 2 (76%) compared to others (Fig. 2c). Mutations in *rpoB* codons 430, 435, 445, and 452 (disputed mutations) reduce the growth rate of M. tuberculosis resulting in their classification as susceptible by MGIT 960 although they confer clinical resistance (18). The frequency of these mutations was highest in lineage 1 (49%) and lowest in lineage 2 (20%) (Fig. 2c). Of the 62 isolates that were phenotypically susceptible to rifampicin but had a resistance-conferring mutation, 32 (51%) belonged to lineage 1 and had at least one of the disputed mutations. In the case of ethambutol, of the 126 phenotypically susceptible isolates with resistance-conferring mutations, 49 (39%), 22 (17%), 40 (32%), 14 (11%) belonged to lineages 1, 2, 3, and 4, respectively, and 1 isolate was identified as mixed lineage (1 + 2). Among the 62 isolates that were phenotypically resistant to pyrazinamide without any known mutation, 49 (79%), 7 (11%), 3 (5%), and 3 (5%) belonged to lineages 1, 2, 3, and 4, respectively. Among the isolates that were phenotypically resistant to streptomycin (*n* = 78) without any known mutation, 39 (50%), 1 (1%), 24 (31%), 14 (18%) were lineage 1, 2, 3, and 4, respectively. Lineage 2 was also observed to be predominant (OFX, 51%; LEV, 44%; and MOX, 49%) among isolates phenotypically resistant to fluoroquinolones.

### Genomic analyses.

A whole genome-based phylogenetic tree was created for 486 of 494 isolates, excluding 8 isolates with a high number of heterozygous sites (see Materials and Methods). This revealed four distinct clusters representing four lineages (Fig. 1a). Lineage 1 displayed higher diversity based on long branch lengths. Pairwise SNP difference was highest for lineage 1, followed by lineage 4, 3, and 2 (Fig. 1b). Of the 34,000 within lineage pairwise comparisons across all 4 lineages, 106 isolate pairs had a SNP difference of ≤10 and were referred to as genetically related. Of these, 91 (86%) pairs belonged to lineage 2, and 10 (9.4%) were lineage 1.

**FIG 3:**
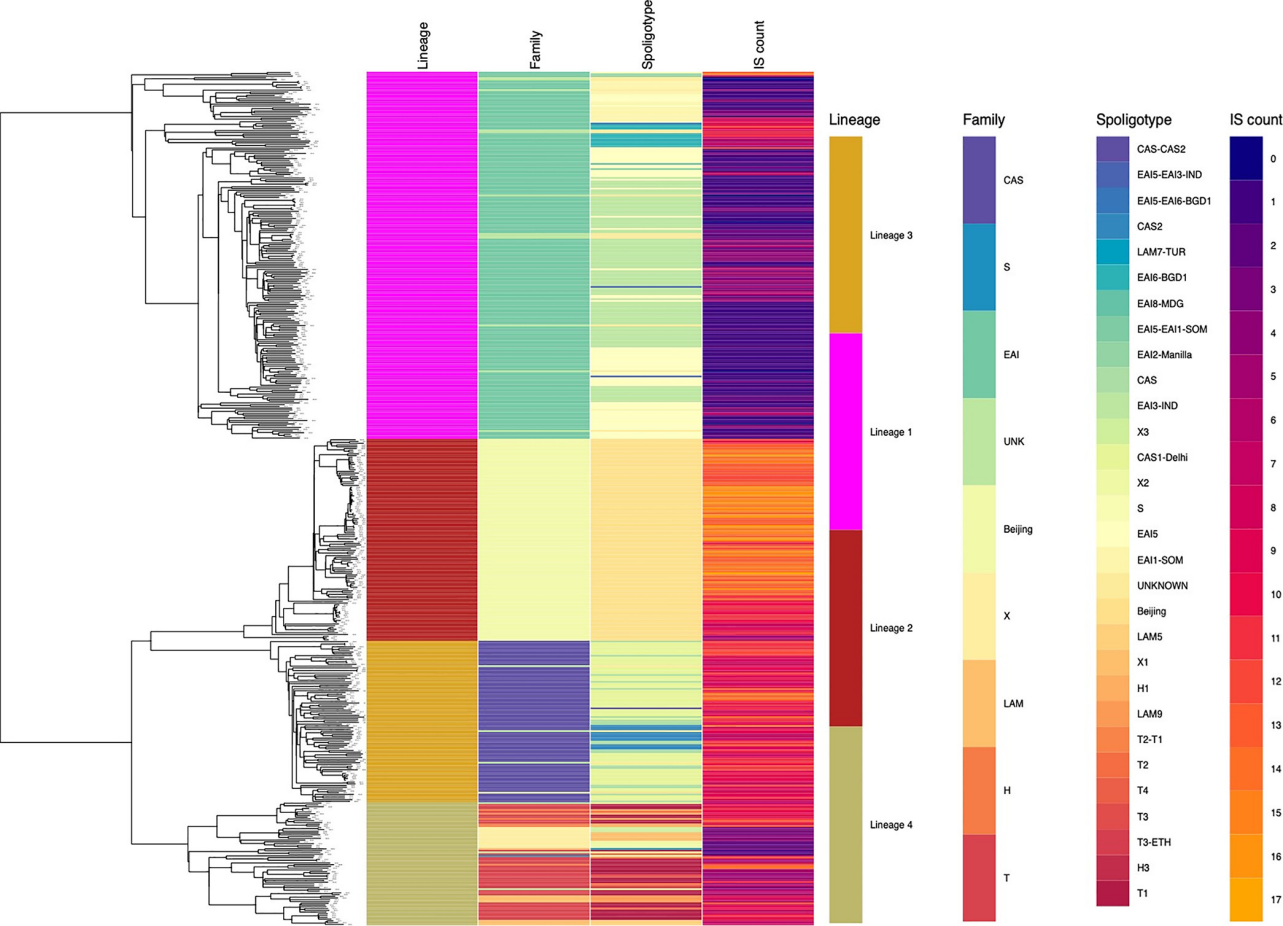
Lineage, digital spoligotype, and *IS*6110 count for 486 study isolates.

Digital spoligotypes were generated from sequence data (Fig. 3). For lineage 1 the EAI family, the predominant spoligotypes were EAI-3-IND (44%, 92/209) followed by EAI-5 (34%, 73/209). Among lineage 3 the CAS family, the predominant spoligotype was CAS1-Delhi accounting for 62% (57/92) of the isolates. Lineage 4 contained several spoligotype families (T, X, S, Latin American-Mediterranean [LAM], and H), of which the spoligotype T family was most frequent (57%, 40/70). Further, among the spoligotype, T family subtype T1 was predominant (90%, 36/40). In previous reports, M. tuberculosis from Southern India was reported to have a low copy number of *IS*6110. We analyzed the number of *IS*6110 insertion sites and their distribution across the study isolates.

Lineage 2 contained the highest number of insertion sites (median of 13), while lineage 1 isolates had the lowest count (median of 1). Further, we observed lineage-specific distribution of these insertion sites across the chromosome in line with the previous reports (19, 20).

## DISCUSSION

Several large-scale studies have shown the potential of WGS-based resistance prediction for the rapid detection of resistance in TB (21–23). Currently, the contribution of sequence data from India in the global databases remains low (7). The predominant lineages (1 and 3) in India contrast with the rest of the world where lineages 2 and 4 dominate. Our study is one of the largest WGS based analyses of drug-resistant M. tuberculosis isolates from a high-burden TB setting, India.

Lineage 1 predominated in our collection from south India, consistent with previous reports (23). Lineage 2 (Beijing lineage) was more associated with resistance, as described previously (24). Overall concordance between phenotype and the genotype-based prediction ranged from 91.7% (INH) to 73.3% (RMP) for first-line drugs and between 97.4% (aminoglycosides) to 82.6% (ethionamide). Sensitivity for INH and RMP was 92% and 98%, respectively, in agreement with previous reports (25). p.S315T *katG* was the predominant mutation, followed by the n.C-15T *fabG1* promoter mutation, consistent with earlier findings from India (9, 10). Interestingly, in our study, most lineage 2 isolates with dual mutation of p.S315X *katG* and the *fabG1* promoter had an association with pre-XDR-TB. We observed 36 phenotypically resistant isolates without any resistance-associated mutation, indicative of uncharacterized mutations or laboratory error. For rifampicin-resistant isolates, the predominant mutation p.S450L was most prevalent among lineage 2 isolates. In contrast, isolates in lineages 1, 3, and 4 had a higher proportion of disputed mutations (mutations in codon 430, 435, 445, and 452), highlighting the importance of defining genetic determinants prevalent in specific settings. We also observed that for the streptomycin resistance phenotype, the current list of genetic determinants did not perform well in identifying resistance, especially for isolates of lineages other than lineage 2. This potentially highlights the knowledge gaps that exist in the global databases due to the low representation of genomic data from India.

The presence of p.A90V and p. D94X mutations in *gyrA* (known to confer resistance to fluoroquinolones) is strongly associated with resistance (26). These were identified in susceptible isolates, suggesting that the critical concentrations used in the phenotypic testing were higher (MOX, 0.5 μg/mL). This is further supported by the recent revision that reduced the critical concentrations, particularly for MOX to 0.25 μg/mL (27). The study included anti-TB drugs PZA, EMB, and ETH for which failures in phenotypic testing are relatively common (28), which could be one of the reasons for lower sensitivities observed for these drugs here.

In a recent study from Thailand, lineage 2 was suggested to be responsible for the increased incidence of MDR-TB (29) due to its association with resistance and increased transmission potential. Our phylogenetic analysis together with pairwise SNP difference suggested the lineage 2 isolates in our collection were more genetically related compared to others. Lineage 2 isolates also had the highest number of genetically related pairs (pairwise distance <10 SNPs). This could potentially indicate the transmission potential of lineage 2 as suggested in previous studies (30). Another possibility could be that these isolates were part of an outbreak, but since this was an archived collection without epidemiological data, we could not investigate further.

*IS*6110 is specific to M. tuberculosis and has been shown to have a role in host adaptation and survival in adverse growth conditions (31). Isolates from India (particularly lineage 1) have a low copy number of *IS*6110. In our data, we observed a lineage-specific pattern for both the number of copies and the distribution of IS6110 insertion sites across the genome. This may suggest a host-specific adaptation strategy, a suggestion that requires further studies to explore its impact on the virulence and transmission of these lineages.

In summary, using the whole genome sequencing data of archived isolates we have confirmed a higher predictive ability of known genetic determinants to identify phenotypic resistance for first-line drugs. We also observed potential lineage-specific patterns in the distribution of resistance-conferring mutations for rifampicin and ethambutol. The study highlights the importance of understanding the local population structure and its association with resistance. The findings from this study warrant the need to consider implementing Whole-genome sequencing to complement phenotypic drug susceptibility testing and together this can provide additional information on the epidemiology, transmission, and spread of M. tuberculosis.

## MATERIALS AND METHODS

### Bacterial isolates.

M. tuberculosis isolates were retrieved from frozen archived collections stored at −80°C at the Indian Council of Medical Research (ICMR)-National Institute for Research in Tuberculosis (NIRT), India, which had originally been isolated between 2014 and 2016 from the state of Tamil Nadu, Telangana, Andhra Pradesh, Andaman and Nicobar Islands, West Bengal and Gujarat. The collection was enriched for drug resistance as these isolates were from patients with confirmed or suspected MDR-TB (failure to first-line anti-TB drug treatment) and submitted to ICMR-NIRT for phenotypic 2nd line drug susceptibility testing. Isolates were recovered using the Lowenstein Jensen (LJ) medium, after which the culture was further amplified on four LJ mediums. The same generation of the sample was used for phenotypic testing and sequencing.

### Phenotypic drug susceptibility testing (DST).

Phenotypic DST was performed using the WHO-endorsed proportion method in an automated mycobacterial growth indicator tube (MGIT) (BD, Franklin Lakes, NJ, USA). Drug susceptibility testing for 12 drugs: isoniazid (INH), rifampicin (RMP), streptomycin (STR), ethambutol (EMB), pyrazinamide (PZA), ofloxacin (OFX), levofloxacin (LEV), moxifloxacin (MOX), kanamycin (KAN), capreomycin (CPR), amikacin (AMIK), and ethionamide (ETH).

### Whole-genome sequencing.

Genomic DNA was extracted from clinical isolates using the CTAB (cetyltrimethylammonium bromide) method (32) and purified using the Genomic DNA Clean and Concentrator kit (Zymo Research, Irvine, CA, USA). Purified DNA was assessed for quality and quantity using Nano DropTM and QubitTM dsDNA assay kit method (ThermoFisher Scientific, Waltham, MA, USA). Sequencing libraries were prepared using the NEBNext Ultra DNA Library preparation kit. In brief, fragmented DNA was subjected to a series of enzymatic steps for repairing the ends and tailing with dA-tail followed by ligation of adapter sequences. Adapter ligated fragments were cleaned up using SPRI beads, and the clean fragments were indexed using limited cycle PCR to generate final libraries for paired-end sequencing on a HiSeq X 10 sequencer (Illumina, San Diego, Ca, USA).

### Sequence-based resistance prediction.

Reads that were at least 30 bp long (150 bp read length) and minimum base quality of 20 were filtered using Trimmomatic v0.36 (33). Contamination with other species was checked using Kraken v1.0 (34). Reads were mapped to the H37Rv reference genome (NC_000916.3) using bwa v0.7.12 (35) using default parameters. Mapping at indels was corrected using picard v2.2.4 (http://broadinstitute.github.io/picard/) and GATK v3.5 (https://gatk.broadinstitute.org/hc/en-us). Variants were identified using samtools v1.3.1 with default parameters. Variants were filtered based on the following metrics: base quality >50, mapping quality >30, read depth >5 and at least one read mapping in either direction. Variants supported by >80% of the mapped reads were classified as homozygous sites and those with <80% mapped reads were classified as heterozygous sites. Variants were compared to a database of resistance-conferring variants generated by combining reports from previous studies (35–38). Lineages were predicted using both SNP-based variants (39) and region of difference (RD) analysis using the tool RD-analyzer (39). Repeat phenotypic testing or sequencing was not performed in the event of discrepancies.

### Genomic analyses.

A pseudogenome was generated for each isolate by substituting the nucleotide base in the H37Rv reference genome sequence with the variants detected using a python script (https://doi.org/10.6084/m9.figshare.11828313.v1). Repetitive regions (29) were masked using bedtools v2.27.1. SNP-sites (40) v2.5.1 were used to identify variable sites from the concatenated alignment of pseudogenomes. The output generated was then used to identify pairwise SNP differences using SNP-dists v0.6.2 (https://github.com/tseemann/snp-dists). The phylogenetic tree was generated from SNP-sites output using RAxML (41) with a GTR-GAMMA model and 1000 bootstrap replications. Genotype and phenotype data were mapped onto the tree using phandango (42). Spoligotypes were predicted from the mapped alignment using the tool lorikeet (http://genomeview.org/jenkins/lorikeet/). *IS*6110 insertion sites were identified using the ISMapper tool (PMID: 26336060). To identify mixed infection, we used a count of heterozygous sites excluding those identified in resistance-conferring genes and repetitive regions. Of the 494 isolates, 486 (98%) had <30 heterozygous sites, the threshold used for mixed infection. Further details are available in the Supplemental Material and Fig. S1. Of the 8 isolates with >30 heterozygous sites, 3 were also identified as mixed based on the detection of different lineages in the phylogenetic SNP-based analysis. These 8 isolates were removed following initial phylogenetic analysis, although the lineages involved in the 3 mixed cases where this could be determined is reported.

### Ethical approval.

This study was approved by the ethical committee of ICMR-National Institute for Research in Tuberculosis, Chennai, India with the no. NIRT-IEC: 2016002 (A).

### Data availability.

The whole-genome sequence for the tuberculous isolate reported in this study is deposited in NCBI (BioProject ID PRJNA822663).
