# Supplementary material for: Mycobacterium tuberculosis Lineages Associated with Mutations and Drug Resistance in Isolates from India
Source: Microbiol Spectr. 2022 Apr 20;10(3):e01594-21. doi: 10.1128/spectrum.01594-21 (PMC9241780; doi:10.1128/spectrum.01594-21)
Supplement: SUPPLEMENTAL FILE 1 — Supplemental material. Download spectrum.01594-21-s001.pdf, PDF file, 0.5 MB [file spectrum.01594-21-s001.pdf]

Supplemental Material for Publication:

Mycobacterium tuberculosis lineages associated with mutations and drug resistance in isolates from India

Siva Kumar Shanmugam[1*], Narender Kumar[2*], Sembulingam Tamilzhalagan[1], Suresh Babu Ramalingam[1], Ashok Selvaraj[1], Udhayakumar rajendhiran[1], Sudha Soliyappan[1], Srikanth P. Tripathy[1], Mohan Natrajan[1], Padmapriyadarshini C[1], Soumya Swaminathan[3], Julian Parkhill[4], Sharon J. Peacock[2], Uma Devi K. Ranganathan[1#]

Identification of mixed samples

Heterozygous sites are those where alignment algorithms cannot assign a particular base. This can occur because of true variation or because of misalignment of reads near repetitive regions. The former can result from mixed infection in a given person, or sample contamination. The use of heterozygous sites to identify mixed infection or contamination has been reported previously for *Mycobacterium tuberculosis* (1) and *Staphylococcus aureus* (2). We assessed mixed infection by considering heterozygous sites that did not occur either in the repetitive regions of H37Rv genome or drug resistance genes. We plotted the absolute count of heterozygous sites for all 498 isolates (figure S1). A minority of isolates had >100 heterozygous sites. Isolates with <50 heterozygous sites were re-plotted (figure S2). From this, we decided on a threshold of 30 as this represented thee 98th percentile in our 486 isolates. All isolates with >30 heterozygous sites were excluded from further analysis.

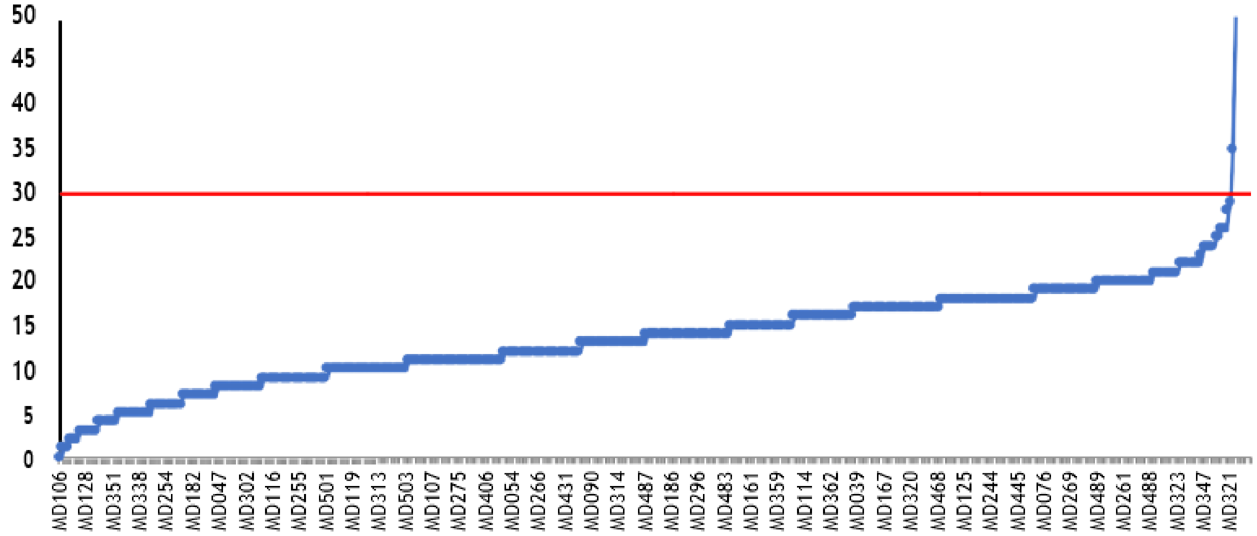

Figure S1: Number of heterozygous sites detected for each isolate in the collection.

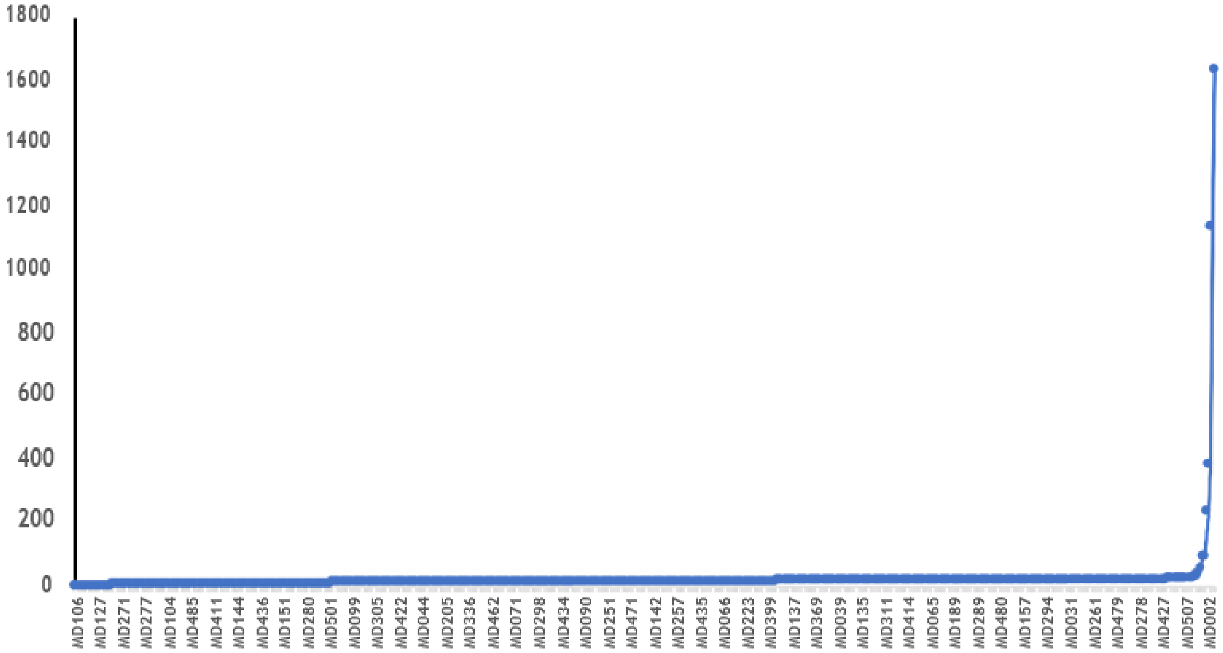

Figure S2: Isolates with <=50 heterozygous sites. The red horizontal line represents the 30-sites cut-off used in the study.

Table S1: The phenotype and the genotype comparison for all the 494 isolates without contamination with other species. In the columns for the anti-TB drugs (columns 3-14), the phenotype is represented by the first letter (R/S) and the letter after the underscore ("_") represents the genotypic prediction (R/S) and in cases where genotypic prediction is R, the genetic mutation identified is listed after the colon(":").

Table S2: The list of the genes mutations which were included in the database used for resistance prediction.

Reference:

1. **Sobkowiak B, Glynn JR, Houben RMGJ,et al; . Identifying mixed Mycobacterium tuberculosis infections from whole genome sequence data. BMC Genomics. 2018 Aug 14;19(1):613.**

2. **Raven KE, Blane B, Kumar N, et al; . Defining metrics for whole-genome sequence analysis of MRSA in clinical practice. Microb Genom. 2020 Apr;6(4):e000354.**
